# Klippel–Trenaunay and Sturge–Weber overlap syndrome with phakomatosis pigmentovascularis

**DOI:** 10.4103/1817-1745.76113

**Published:** 2010

**Authors:** Monika Chhajed, Sadbhavna Pandit, Neeraj Dhawan, Amit Jain

**Affiliations:** Department of Pediatrics, Government Medical College, Sec 32, Chandigarh - 160 032, India; 1 Department of Radiology, Government Medical College, Sec 32, Chandigarh - 160 032, India

**Keywords:** Klippel–Trenaunay syndrome, phakomatosis, Sturge–Weber syndrome

## Abstract

Klippel–Trenaunay syndrome and Sturge–Weber syndrome are rare disorders with neurologic and cutaneous signs of vascular origin. Phakomatosis pigmentovascularis represents the association of widespread, aberrant, and persistent nevus flammeus and pigmentary abnormalities. We describe a case with features suggestive of overlap between them. A ten-month-old boy presented with seizures, developmental delay, skin lesions on face, trunk and legs, buphthalmos and right lower limb hypertrophy. CT scan of head showed atrophy of brain and calcification. Our case had overlap of Klippel–Trenaunay syndrome and Sturge–Weber syndrome with phakomatosis pigmentovascularis

## Introduction

Sturge–Weber syndrome occurs sporadically with a frequency of 1 in 50,000. It is characterized by meningofacial angiomatosis with cerebral calcification.[1] Klippel-Trenaunay syndrome is characterized by a triad of cutaneous vascular malformation along with bony or soft tissue hypertrophy and venous varicosities. Phakomatosis pigmentovascularis is a distinctive association of cutaneous hemangiomas and melanocytic nevi.

## Case Report

A ten-month-old male child presented with developmental delay, reduced vision and right-sided focal seizures. A similar episode of seizure was reported one month back. He was the first born child of nonconsanguineous marriage. There was no significant family or birth history. The developmental milestones were delayed.

**Figure 1:**
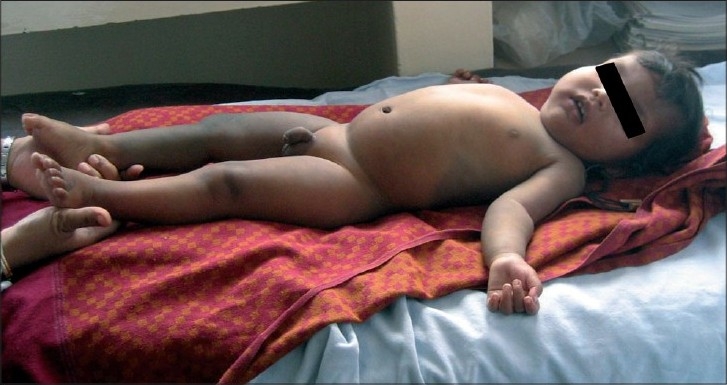
Showing hyperpigmented lesion on right leg and trunk.

On examination, his weight was 9.3 kg (25^th^ percentile), height was 72 cm (25^th^ percentile) and head circumference was 52 cm. (>95^th^ percentile, macrocephaly). Developmental assessment revealed a global developmental delay as he could not sit without support, had an absent palmar grasp, monosyllabic speech, would not smile at mirror image, or play with ball. There was a port-wine stain on his face and hyperpigmented lesions over trunk, abdomen and right leg as seen in Figure 1 suggestive of dermal melanocytosis. Hyperpigmentation on the left cornea and facial skin were suggestive of oculocutaneous melanosis (nevus of Ota). He had megalocornea and bilateral buphthalmos. His right lower limb was hypertrophied. Calf circumferences of right and left lower limb were 20.6 cm and 18.5 cm, respectively, thigh circumference of right and left lower limb were 26.5 cm and 25.3 cm, respectively, foot length of right and left lower limb were 137.91 cm and 124.21 cm, respectively.

**Figure 2:**
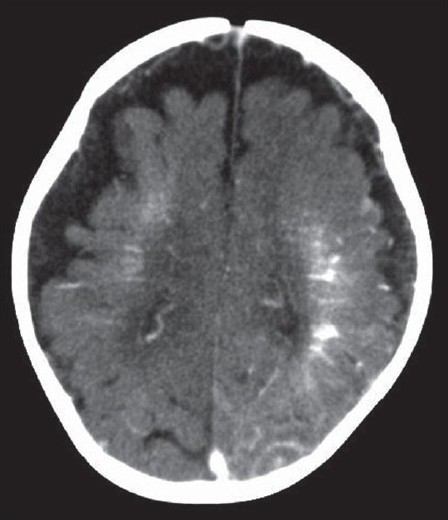
CT of head showing atrophy of brain and calcification of left parietal region.

The computerized tomography (CT) scan of brain of the patient as seen in Figure 2 showed generalized atrophy of brain along with generalized hyper dense area in white matter mainly in frontal lobe and left parietal lobe. Dystrophic calcification suggestive of chronic congenital ischemic change due to venous malformation was also noted. There was mild post contrast enhancement in the left parietal-occipital region. Ultrasound of abdomen did not show any vascular and renal anomaly. As the child had both the features of Sturge–Weber syndrome and Klippel–Trenaunay syndrome, a diagnosis of an overlap syndrome was made.

## Discussion

**Figure 3:**
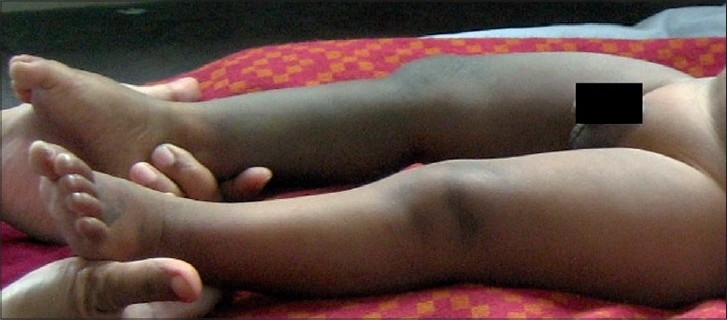
Showing leg hypertrophy

Sturge–Weber syndrome is a mesodermal phakomatosis characterized by port-wine naevus covering face and cranium supplied by first division of trigeminal nerve along with atrophy and calcification of cerebral hemisphere homolateral to the skin lesion.[1] This syndrome occurs sporadically with a frequency of approximately 1 in 50,000.[2] Sturge–Weber syndrome is associated with glaucoma, seizures, paresis, and neurodevelopmental delay. About 70% of patients with epilepsy have their first seizure within the first year of their life. Neuroradiological findings in these patients show leptomeningeal enhancement along with signs of cortical atrophy and calcifications in the subjacent area.[3] In our patient, the central nervous system abnormalities on CT scan along with the port-wine stain, glaucoma, seizures, and developmental delay pointed to Sturge–Weber syndrome. But the right leg hypertrophy, extensive cutaneous vascular lesions and macrocephaly could not be explained by Sturge-Weber syndrome alone. Klippel–Trenaunay syndrome is a rare entity characterized by the combination of capillary nevus, early onset of varicosities and hypertrophy of tissues and bones of the affected limb.[4] Cutaneous lesions can occur in any area, but are more commonly located on the legs, buttocks, abdomen and lower trunk. The varicosities in Klippel–Trenaunay syndrome appear in most patients by the age of 12 years.[3] Central nervous system abnormalities associated with Klippel–Trenaunay syndrome include microcephaly, macrocephaly, cerebral arteriovenous malformations, spinal arteriovenous malformations, and orbito-frontal varices.[1] Our patient had leg hypertrophy associated with ipsilateral cutaneous vascular malformations and macrocephaly, which are features of this syndrome[Figure 3].

Phakomatosis pigmentovascularis is characterized by the association of extensive nevus flammeus and pigmentary nevi in the form of widespread, aberrant and persistent Mongolian spots.[5] There are four types of phakomatosis pigmentovascularis all having nevus flammeus as a component. Along with nevus flammeus, type I has an epidermal nevus, type II has blue spots with or without nevus anemicus, type III has nevus spilus, with or without nevus anemicus, type IV has blue spots and nevus spilus with or without nevus anemicus.[6] The findings in our patient are in accordance with type IIa, according to the classification of phakomatosis pigmentovascularis.

Over 40 reports of patients with overlap of Klippel–Trenaunay syndrome and Sturge–Weber syndrome have been published.[3] Neuroradiological studies have been performed only in few such studies.[2,3,7,8] The CT scan abnormalities of central nervous system noted in them were malformation of the circle of Willis, cerebral hemihypertrophy, aplasia of the cervical internal carotid artery, cerebral atrophy, prominent choroid plexus, cerebral calcifications, leptomeningeal enhancement. Only in few patients, typical intracranial findings as seen in Sturge-Weber syndrome were seen. There is no specific curative treatment for this combined disorder. The management is directed towards underlying systemic and local anomalies. Our case represented an overlap syndrome between Klippel-Trenaunay syndrome and Sturge-Weber syndrome in association with phakomatosis pigmentovascularis, which to the best of our knowledge has been reported in very few earlier studies[6,7,9] and has not been reported from India.
